# NFKB1 gene rs28362491 polymorphism is associated with the susceptibility of acute coronary syndrome

**DOI:** 10.1042/BSR20182292

**Published:** 2019-04-17

**Authors:** Si-Yu Jin, Jun-Yi Luo, Xiao-Mei Li, Fen Liu, Yi-Tong Ma, Xiao-Ming Gao, Yi-Ning Yang

**Affiliations:** 1 Department of Cardiology, First Affiliated Hospital of Xinjiang Medical University, Urumqi, Xinjiang, China; 2 Xinjiang Key Laboratory of Cardiovascular Research, Urumqi, Xinjiang, China; 3 Baker IDI Heart and Diabetes Institute, Department of Surgery, Central Clinical School, Monash University, Melbourne, Victoria, Australia; 4 Xinjiang Key Laboratory of Medical Animal Model Research, Urumqi, Xinjiang, China

**Keywords:** acute coronary syndrome, NFKB1 gene, polymorphism, susceptibility

## Abstract

The acute coronary syndrome (ACS) is a complex disease where genetic and environmental factors are involved. NF-κB, a central regulator of inflammation, is involved in various inflammatory diseases. The aim of the present study was to explore the association between NFKB1 gene rs28362491 (-94ATTGins/del) polymorphism and ACS. A total of 778 ACS patients and 1112 healthy subjects were included in our study. The TaqMan SNP genotyping assays was used to analyze the rs28362491 polymorphism. The lesion extent of coronary artery was assessed by Gensini Score and lesion vessel number in ACS patients. For total and males, the frequencies of the mutant DD genotype and D allele were significantly higher in ACS patients than that in control subjects (total: DD genotype: 18.0 vs 14.1%, *P*=0.009, D allele: 43.0 vs 37.9%, *P*=0.002, males: DD genotype: 20.6 vs 15.3%, *P*=0.042, D allele: 44.2 vs 38.8%, *P*=0.013). After multivariate logistic regression analysis, we found that individuals with mutant DD genotype had 1.329-fold higher risk of ACS compared with individuals with ID and II genotypes. Moreover, ACS patients with DD genotype were worse stenosis of coronary artery compared with patients carrying II or ID genotype. In conclusion, our study demonstrated that the mutant DD genotype of NFKB1 gene was associated with the risk and severity of ACS in Han population in Xinjiang, northwest of China.

## Introduction

ACS is a syndrome due to decreased blood flow in the coronary arteries such that part of the cardiomyocyte is unable to function properly or dies. Coronary atherosclerosis is the underlying condition for ACS. Inflammation is occupying a prominent position in the initiation and progression of atherosclerosis, implicating the involvement of inflammatory cytokines in the atherosclerotic processes.

NF-κB, which is a prominent inducible transcription factor that regulates the immune system, consists of a total of five proteins that are predominantly present in an inactivated state in the cytosol as either homo- or heterodimers that include: RelA (p65), RelB, c-Rel, p105, and p100. The p105 must undergo post-translational processing through the proteasome to form the active subunits, p50. The NFKB1 gene located on the human chromosome 4q24 and consisted of 24 exons. The functional polymorphism in the promoter region of NFKB1 gene is the four bases of ATTG insertion/deletion variant (-94ins/del ATTG, rs28362491), encodes three genotypes: wild-type homozygous insertion (ins/ins, II genotype), variant homozygous deletion (del/del, mutant DD genotype), and heterozygous (ins/del, ID genotype). Previous studies have shown that the rs28362491 (-94ATTGins/del) polymorphism of the NFKB1 gene was associated with many inflammatory diseases such as Grave’s disease, ulcerative colitis, and systemic lupus erythematosus [4–6]. The rs28362491 (-94ATTGins/del) polymorphism of the NFKB1 gene has been reported in shear-induced eNOS gene expression and endothelial dysfunction in prehypertension and stage I hypertension, mostly due to differences in NFKB1 gene transcriptional activity. These evidences suggest that NFKB1 gene mutant DD genotype may be a functional mutant in cardiovascular diseases [7–12]. Our previous study reported that mutant DD genotype of NFKB1 gene in HUVECs reduced total p50 subunit expression versus wild-type cells and it may be the mechanism of NFKB1 gene polymorphism increase the risk and severity of coronary artery disease (CAD) [13]. However, its relationship with ACS remains unclear.

Thus, in the present study, the relationship between the rs28362491 (-94ATTGins/del) polymorphism of the NFKB1 gene and ACS was investigated in patients with ACS and control subjects in a Chinese Han population.

## Materials and methods

### Ethical approval

The present study was approved by the Ethics Committee of the First Affiliated Hospital of Xinjiang Medical University and conducted according to the standards of the Declaration of Helsinki. Written informed consent was obtained from each participant.

### Subjects

The subjects were Chinese Han population who lived in Xinjiang, northwest of China, and they were recruited from the First Affiliated Hospital of Xinjiang Medical University from 2010 to 2014. Written informed consent was obtained from all participants. The diagnosis of ACS was based on typical electrocardiographic and enzymatic criteria and confirmed by echocardiography and coronary angiography, according to ESC guidelines. All of the control subjects also underwent a coronary angiogram to confirm no stenosis in their coronary arteries. Moreover, they did not show clinical or electrocardiogram evidence of ACS. Frankly, the controls were not healthy individuals, some of them have hypertension, some of them have DM, and some of them have hyperlipidemia, which means control group expose to the same risk factors of CAD while the results of coronary angiogram are normal. For all of the subjects, the exclusion criteria were these who with concomitant valvular heart disease, congenital heart disease, and/or non-ischemic cardiomyopathy.

### Genetic analysis

Genomic DNA was purified from total leukocytes in peripheral blood using the TIANamp Genomic DNA Kit (Tiangen Biotech, China), which were concentrated 50 ng/μl and stored at −80°C for future analysis.

We used the TaqMan SNP genotyping assay (Applied Biosystems, Foster City, CA, U.S.A.) to genotype the rs28362491 (-94ATTGins/del) polymorphism of the NFKB1 gene. The primers and probes used in assay were chosen according to the information at the ABI website (http://myscience.appliedbiosystems.com). Applied Biosystems 7900HT standard real-time PCR (PCR) system was used for the DNA amplification. The results of each polymorphism of NFKB1 gene were read by the sequence detection systems automation controller software v2.3 (ABI). The reaction system of PCR amplification was as follows: 3 μl of TaqMan Universal Master Mix, 0.12 μl probes, and 1.88 μl ddH_2_O in a 6 μl final reaction volume containing 1 μl DNA (50 ng). Amplification cycling conditions were as follows: 95°C for 5 min, 40 cycles of 95°C for 15 s, and 60°C for 1 min. Samples with ambiguous genotypes that were not separated by discrete clusters were regenotyped.

### Characteristic data collection

Characteristic data (age, smoking and drinking habits and medical history) were collected using questionnaires, and height, body weight and blood pressure were measured by doctors. Participants, who had smoked at least one cigarette per day in the previous 12 months, were considered as current smokers. Fasting peripheral blood samples were obtained from all participants for the assessment of routine biochemical variables. Essential hypertension (EH) was defined as systolic blood pressure ≥140 mmHg and/or diastolic blood pressure ≥90 mmHg on at least two separate occasions, or antihypertensive treatment. Total cholesterol (TC), low density lipoprotein-cholesterol (LDL-C), high density lipoprotein-cholesterol (HDL-C), and triglyceride (TG) were measured by standard enzymatic methods using Dimension AR/AVL Clinical Chemistry System (DADE Bchring, Newark, NJ, U.S.A.) in the Central Laboratory of the First Affiliated Hospital of Xinjiang Medical University.

### Coronary angiography

Coronary angiography was performed in all ACS patients and controls. Gensini score: angiographic stenosis of a culprit artery in the range of 0–25% was scored as 1 point, stenosis in the range of 25–50% was scored as 2 points, 50–75% was scored as 4 points, 75–90% was scored as 8 points, 90–99% was scored as 16 points, and total occlusion was scored as 32 points. A multiplier was assigned to each main vascular segment based on the functional significance of the myocardial area supplied by that segment: 5 for the left main coronary artery, 2.5 for the proximal segment of the left anterior descending (LAD) coronary artery and the proximal segment of the circumflex artery, 1.5 for the mid-segment of the LAD, 1.0 for the right coronary artery, the distal segment of the LAD, mid-distal region of the circumflex artery, the posterolateral artery and the obtuse marginal artery, and 0.5 for other segments. Angiographic evaluations were reviewed by two independent interventional cardiologists blinded to the study information. In case of disagreement, the decision was based on the judgment of the third, more experienced cardiologist.

### Statistical analysis

All statistical analyses were performed by SPSS 17.0 software (SPSS Institute, Chicago, IL, U.S.A.). All continuous variables were expressed as mean ± standard deviation, and categorical data are shown as percentages (%). The *P* value of the continuous variables was calculated by the independent *t* test. The *P* value of the categorical variables was calculated by chi-square test. Logistic regression analysis was used to assess the contribution of the major risk factors to ACS. *P<*0.05 were deemed statistically significant.

## Results

### Clinical characteristics of control subjects and ACS patients

A total of 1890 individuals (778 ACS patients and 1112 healthy controls) were recruited in the present study. The clinical characteristics of the individuals are shown in Table 1. The concentrations of age, TC, LDL-C, TG and the percentages of diabetes, LVEF and BMI, were significantly higher in ACS patients than that in controls. In addition, the plasma concentrations of HDL and the percentages of smoking and hypertension were significantly lower in ACS patients compared with controls (all *P*<0.05). There were no differences between the groups with respect to sex, DBP, and SBP.

**Table 1:** Clinical characteristics of control subjects and ACS patients

Characteristics	Control (n = 1112)	ACS (n = 778)	*P* value
Female n(%)	485 (43.6%)	341 (43.8%)	0.926
Age (years)	58.47±11.21	59.50 ± 10.06	0.038*
Smoking n(%)	324 (29.1%)	267 (34.3%)	0.017*
Hypertension n(%)	439 (39.5%)	404 (51.9%)	<0.001**
Diabetes, n(%)	144 (12.9%)	219 (28.1%)	<0.001**
BMI (kg/m^2^)	25.73 ± 3.17	25.79 ± 2.89	0.014*
SBP (mmHg)	125.56 ± 17.66	126.80 ± 18.72	0.199
DBP (mmHg)	78.64 ± 11.19	77.28 ± 11.34	0.356
TG (mmol/l)	1.55 ± 0.58	1.59 ± 0.55	0.032*
TC (mmol/l)	4.42 ± 0.64	4.54 ± 0.74	<0.001**
HDL-C (mmol/l)	1.07 ± 0.22	1.03 ± 0.20	0.001*
LDL-C (mmol/l)	2.36 ± 0.54	2.46 ± 0.61	<0.001**
LVEF (%)	64.78 ± 6.41	61.48 ± 7.74	0.02*

HbA1c, glycosylated hemoglobin.

*P<0.05, **P<0.001

### The frequencies of the mutant DD genotype and D allele were significantly higher in ACS patients than that in the control group

Table 2 shows the distribution of alleles and genotypes of rs28362491 for the NFKB1 gene. For total, the frequencies of the mutant DD genotype and D allele were significantly higher in ACS patients than that in control subjects (DD genotype: 18.0 vs 14.1%, *P*=0.009, D allele: 43.0 vs 37.9%, *P*=0.002). For males, the frequencies of DD genotype and D allele were also higher in ACS patients than that in control subjects (DD genotype: 20.6 vs 15.3%, *P*=0.042, D allele: 44.2 vs 38.8%, *P*=0.013). Unfortunately, we did not found any difference of the frequencies of DD genotype and D allele in females. For the total population, the distribution of the dominant model (II vs ID+DD) and the recessive model (DD vs II+ID) were significantly different between ACS patents and controls (for dominant model: *P*=0.007; for recessive model: *P*=0.023).

**Table 2:** Distribution of NFKB1 -94 ATTG ins /del mutant in control subjects and ACS patients

	Total			Males			Females		
	Control n (%)	ACS n (%)	*P* value	Control n (%)	ACS n (%)	*P* value	Control n (%)	ACS n (%)	*P* value
Allele									
I	1381 (62.1%)	889 (57.0%)			768 (61.2%)	488 (55.8%)		613 (63.2%)	401 (58.8%)
D	843 (37.9%)	671 (43.0%)	0.002*	486 (38.8%)	386 (44.2%)	0.013*	357 (36.8%)	281 (41.2%)	0.071
Genotyping									
II	426 (38.3%)	251 (32.3%)		237 (37.8%)	141 (32.3%)		189 (39.0%)	110 (32.3%)	
ID	529 (47.6%)	387 (49.7%)	0.009*	294 (46.9%)	206 (47.1%)	0.042*	235 (48.5%)	181 (53.1%)	0.136
DD	157 (14.1%)	140 (18.0%)		96 (15.3%)	90 (20.6%)		61 (12.6%)	50 (14.7%)	
Dominant model									
II	426 (38.3%)	251 (32.3%)		237 (37.8%)	141 (32.3%)		189 (39.0%)	110 (32.3%)	
ID+DD	686 (61.7%)	527 (67.7%)	0.007*	390 (62.2%)	296 (67.7%)	0.064	296 (61.0%)	231 (67.7%)	0.048*
Recessive model									
DD	157 (14.1%)	140 (18.0%)			96 (15.3%)	90 (20.6%)		61 (12.6%)	50 (14.7%)
II+ID	955 (85.9%)	638 (82.0%)	0.023*	531 (84.7%)	347 (79.4%)	0.076	424 (87.4%)	291 (85.3%)	0.387
Additive model									
ID	529 (47.6%)	387 (49.7%)		294 (46.9%)	206 (47.1%)		235 (48.5%)	181 (53.1%)	
II+DD	583 (52.4%)	391 (50.3%)	0.353	333 (53.1%)	231 (52.9%)	0.936	250 (51.5%)	160 (46.9%)	0.191

The *P* value of categorical variable was calculated using chi-square test.

* *P*<0.05.

### NFKB1 gene mutant DD genotype was associated with a higher risk of ACS

Multifactor logistic regression analysis revealed seven independent risk factors for ACS: mutant DD genotype, age, smoking, EH, DM, TC, HDL-C, and LDL-C. After adjustments of smoking, EH, DM, TC, HDL-C, and LDL-C, patient with mutant DD genotype, had a significantly higher risk of ACS (OR = 1.329; 95% CI: 1.027–1.720; *P*=0.031) (Table 3).

**Table 3:** Multivariate logistic regression for ACS patients

Risk factor	β	SE	Waldχ^2^	*P* value	OR	95% CI
DD vs II+ID	0.284	0.132	4.674	0.031*	1.329	1.027–1.720
Age	0.007	0.005	2.208	0.137	1.007	0.998–1.061
Smoking	0.232	0.104	4.943	0.026*	1.261	1.028–1.546
EH	0.407	0.098	17.068	<0.001**	1.502	1.238–1.821
DM	0.874	0.122	51.069	<0.001**	2.396	1.886–3.046
TC	0.194	0.075	6.738	0.009*	1.214	1.049–1.405
HDL-C	-−0.630	0.234	7.278	0.007*	0.532	0.337–0.842
LDL-C	0.201	0.09	5.049	0.025*	1.223	1.026–1.458

DM, diabetes mellitus; EH, essential hypertension; OR, odds ratio; SE, standard error.

*P<0.05, **P<0.001.

### Mutant DD genotype was associated with worse stenosis of coronary artery

**Figure 1:**
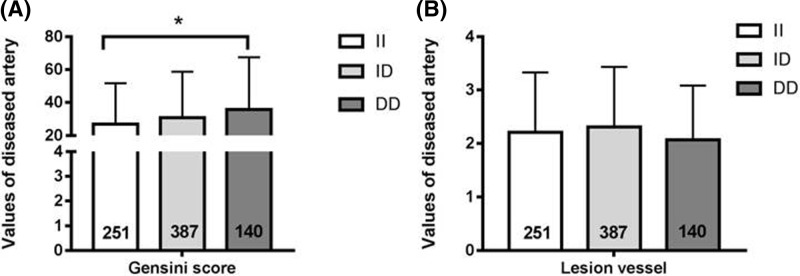
Influence of the NFKB1 gene DD mutant on lesion extent of coronary artery Gensini score (**A**) and lesion vessel number (**B**) in ACS patients. n = 778 (II genotype n = 251, ID genotype n = 387, DD genotype n = 140). Values are means ± standard deviation. *P<0.05.

The Gensini score was significantly higher in ACS patients with DD genotype than that in patients with II genotype (*P*<0.05, Figure 1). After general linear model analysis adjusted for age, smoking, EH, DM, TC, HDL-C, and LDL-C, we found that the difference was also remaining amongst ACS patients with different genotypes. However, the number of multivessel disease was not significant difference in ACS patients with DD genotype compared with ID or II genotype.

## Discussion

In the present study, we found that variation in the NFKB1 gene is significantly associated with ACS. Individuals with the mutant DD genotype had a higher risk and severity of ACS compared with individuals with II or DD genotypes. Moreover, ACS patients with mutant DD genotype were worse stenosis of coronary artery compared with patients carrying II or ID genotype.

The NFKB1-94ins/del ATTG polymorphism has been extensively studied in cardiovascular diseases. However, so far contrasting results have been reported. Vogel et al. [15] investigated that carriers of D allele of the functional NFKB1 ATTG ins/del promoter polymorphism are at higher risk of coronary heart disease than homozygous I allele carriers in three independent prospective studies of generally healthy Caucasians. López-Mejías R’s [16] suggested rheumatoid arthritis patients carrying the mutant DD genotype had higher risk of cardiovascular events than those with II genotype. Mishra A [17] found that mutant DD genotype may elevate risk of heart failure by increasing ventricular remodeling and worsening left ventricular function. Hong-Mei Lai [18] indicated that NFKB1-94 ins/del ATTG polymorphism is functionally associated with IL-6 expression, suggesting a mechanistic link between NFKB1-94 ins/del ATTG polymorphism and CAD susceptibility. However, Boccardi V [19] suggested that mutant DD genotype and plasma fibrinogen interaction may contribute to lower myocardial infarction susceptibility by reducing of activated NF-кB. In our study, NFKB1-94ins/del ATTG polymorphism has been analyzed in Chinese Han population. We found that ACS patients had significantly higher distribution of mutant DD genotype compared with controls. Individuals with mutant DD genotype had 1.329-fold higher risk of ACS compared with individuals with ID and II genotypes.

Endothelial dysfunction is an early hall mark of atherosclerosis. Yoshino S [20] indicated that the polymorphism of rs3774933 and rs1599961 in NFKB1 gene were associated with an increased risk of coronary endothelial dysfunction in Caucasus population. Our precious study also reported that mutant DD genotype of rs28362491 in NFKB1 gene was a accompanied with endothelial dysfunction and higher inflammatory status in CAD patients. Moreover, under oxidative stress, mutant DD carrying HUVECs exhibited elevated activation of NF-κB pathway and altered expression of its downstream targetted genes, which lead to more vulnerable to oxidative to apoptosis [13]. In our study, we found that the severity of coronary artery were worse in ACS parents who carries mutant DD genotype. These results indicate that the mutant DD genotype of NFKB1 gene may be an important functional mutant in endothelial function especially in ACS patients. The mechanisms are still not well understood and need further study. However, there are some studies explored the mechanisms of the functional polymorphism in other diseases. In 2004, Karban AS [21] first indicated that NFKB1 -94delATTG allele showed less promoter activity than comparable constructs containing the -94insATTG allele, and this genetic variant was associated with ulcerative colitis. Park JY [22] reported that proteins present in the nuclei of endothelial cells preferentially bound to the I allele NFKB1 promoter compared with the D allele by competitive EMSAs. They also found that the I allele promoter had significantly higher activity than the D allele and homozygous II genotype cells had higher p50 expression levels than homozygous DD genotype cells. Moreover, Chen F [23] reported that the -94delATTG allele promoter of NFKB1 abolished the binding site of transcription factor and it may increase the susceptibility to oral cancer.

In our study, we found the percentage of diabetes was significantly higher in ACS patients than that in controls. The individuals with DM had 2.396-fold higher risk of ACS compared with individuals without DM. As we know, there are numerous factors that intersect in the pathogenesis of CAD such as dyslipidemia, insulin resistance and hyperglycemia, and many of these are either enhanced by or introduced by diabetes [24]. In essence, the diabetic state accelerates most cardiac pathologies due to abnormalities in systemic and local vascular inflammation, endothelial and microvascular injury, altered thrombosis, autonomic nerve dysfunction, and likely membrane instability in nerves, smooth muscle, and endothelium. This leads to deposition of lipids and oxidized lipoproteins in the wall, which induces a macrophage and T-lymphocyte driven immune response. The result is a thickened intima and a vicious cycle of local inflammation and apoptosis, leading to progressive endothelial injury and formation of lipid-rich plaques. It is this process, recurring over time with small micronsults, combined with abnormal platelet reactivity and fibrin deposition in diabetes that predisposes to both progressive luminal narrowing (impaired blood flow and chronic ischemia) and plaque rupture with thrombosis that can block blood flow acutely (myocardial infarction) [25]. Thus, we think that is why the increase in diabetic subjects in the risk versus control populations in our result.

There are several limitations on our study. First, this is a single-center study just in Chinese Han population; thus, our findings still need to be verified in other ethnicities and in a larger population. Second, in the present study, only one variant, rs28362491, was investigated rather than other variants in the NFKB1 gene. As we know, polymorphisms are the most abundant form of genetic variations and have a great potential for mapping genes underlying complex genetic traits. But their effects were microeffect. Much more studies of the other SNPs are needed to investigate the association of ACS and NFKB1 gene.

In summary, the results of present study demonstrate that NFKB1 gene mutant DD genotype is significantly associated with the risk of ACS and the severity of coronary artery in ACS patients. Therefore, screening for this mutant may provide an option for increasing the diagnostic capacity and improving the ACS risk assessment in susceptible individuals.

## Abbreviations

ACS: acute coronary syndrome
CAD: coronary artery disease
DBP: diastolic blood pressure
EH: essential hypertension
eNOS: endothelial nitric oxide synthase
HDL-C: high density lipoprotein-cholesterol
HUVEC: human umbilical vein endothelial cells
LDL-C: low density lipoprotein-cholesterol
LVEF: left ventricular ejection fraction
NF-κB: nuclear factor kappa B
SBP: systolic blood pressure
TC: total cholesterol
TG: triglyceride
